# Supplementary material for: Nradd Acts as a Negative Feedback Regulator of Wnt/β-Catenin Signaling and Promotes Apoptosis
Source: Biomolecules. 2021 Jan 14;11(1):100. doi: 10.3390/biom11010100 (PMC7828832; doi:10.3390/biom11010100)
Supplement: Supplementary file 1 [file biomolecules-11-00100-s001.pdf]

Supplementary Figure for Ozalp et. al.

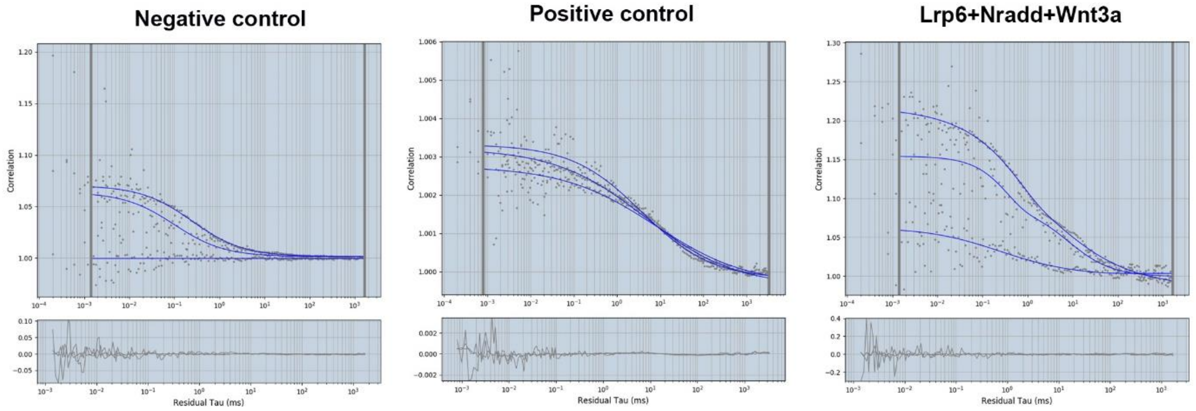

Figure S1 Autocorrelation and cross-correlation analysis of EGFP and mCherry/mRuby3 tagged proteins in live U2OS cells. Representative graphics show low cross-correlation (negative control) and high cross-correlation (positive control and Lrp6+Nradd+Wnt3a). Autocorrelation function (FCS) for GFP (top curve) and mCherry/mRuby3 (middle curve). Cross-correlation function (FCCS, bottom curve) for Nradd-GFP and Lrp-mCherry in the plasma membrane. x axis and y axis show lag time (ms) and correlation G(τ)I, respectively.
